# 1-(3-Benzyl-4,6-dibenz­yloxy-2-hy­droxy­phen­yl)ethanone

**DOI:** 10.1107/S160053681203588X

**Published:** 2012-09-05

**Authors:** Tania N. Hill, C.-M. Kuo, Barend C. B. Bezuidenhoudt

**Affiliations:** a Department of Chemistry, University of the Free State, PO Box 339, Bloemfontein 9300, South Africa

## Abstract

The title compound, C_29_H_26_O_4_, is essentially planar in the acetophenone portion that includes both the hy­droxy and a benz­yloxy O atoms, with an r.m.s. deviation of 0.0311 Å. The other two substituents inter­sect the plane at 70.45 (3) and 59.55 (4)°. In the molecule there is an intramolecular O—H⋯O hydrogen bond. In the crystal, mol­ecules are linked by C—H⋯O hydrogen bonds, as well as C—H⋯π and π-stacking inter­actions, with centroid–centroid distances 3.6570 (2) Å.

## Related literature

For applications of acetophenones, see: Burdock (2010 ▶); Marais *et al.* (2005 ▶).
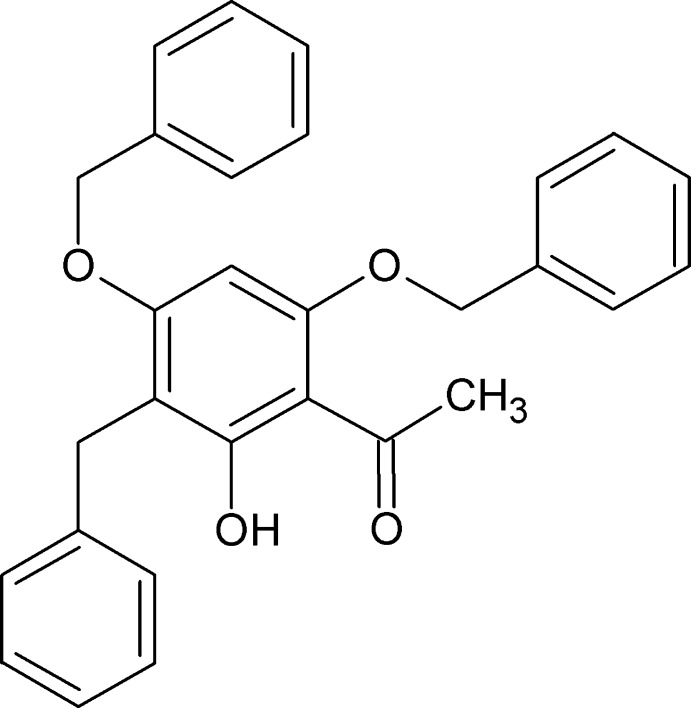


## Experimental

### 

#### Crystal data

C_29_H_26_O_4_
*M* *_r_* = 438.5
Triclinic, 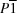

*a* = 8.2349 (5) Å
*b* = 10.5411 (7) Å
*c* = 13.5115 (9) Å
α = 93.412 (3)°
β = 94.139 (3)°
γ = 105.102 (3)°
*V* = 1125.66 (13) Å^3^
*Z* = 2
Mo *K*α radiation
μ = 0.09 mm^−1^
*T* = 100 K
0.39 × 0.12 × 0.03 mm

#### Data collection

Bruker X8 APEXII 4K KappaCCD diffractometer
Absorption correction: multi-scan (*SADABS*; Bruker, 2004 ▶) *T*_min_ = 0.968, *T*_max_ = 0.997
22325 measured reflections
5521 independent reflections
4071 reflections with *I* > 2σ(*I*)
*R*_int_ = 0.032

#### Refinement

*R*[*F* ^2^ > 2σ(*F* ^2^)] = 0.043
*wR*(*F* ^2^) = 0.107
*S* = 1.03
5521 reflections
300 parameters
H-atom parameters constrained
Δρ_max_ = 0.31 e Å^−3^
Δρ_min_ = −0.22 e Å^−3^

### 

Data collection: *APEX2* (Bruker, 2005 ▶); cell refinement: *SAINT-Plus* (Bruker, 2004 ▶); data reduction: *SAINT-Plus*; program(s) used to solve structure: *SHELXS97* (Sheldrick, 2008 ▶); program(s) used to refine structure: *SHELXL97* (Sheldrick, 2008 ▶); molecular graphics: *DIAMOND* (Brandenburg & Putz, 2005 ▶); software used to prepare material for publication: *WinGX* (Farrugia, 1999 ▶).

## Supplementary Material

Crystal structure: contains datablock(s) global, I. DOI: 10.1107/S160053681203588X/ng5288sup1.cif

Structure factors: contains datablock(s) I. DOI: 10.1107/S160053681203588X/ng5288Isup2.hkl

Supplementary material file. DOI: 10.1107/S160053681203588X/ng5288Isup3.cml

Additional supplementary materials:  crystallographic information; 3D view; checkCIF report

## Figures and Tables

**Table 1:** Hydrogen-bond geometry (Å, °) *Cg*1 and *Cg*3 are the centroids of the C1–C6 and C31–C36 rings, respectively.

*D*—H⋯*A*	*D*—H	H⋯*A*	*D*⋯*A*	*D*—H⋯*A*
C56—H56⋯O5	0.95	2.39	2.7282 (15)	101
C67—H67*B*⋯O5	0.99	2.34	2.7882 (15)	106
O1—H1⋯O2	0.84	1.72	2.4744 (13)	148
C57—H57*A*⋯O2^i^	0.99	2.58	3.4592 (16)	148
C23—H23*B*⋯*Cg*3^ii^	0.98	2.83	3.6790 (16)	145
C57—H57*B*⋯*Cg*1^ii^	0.99	2.64	3.5106 (14)	146

Symmetry codes: (i) 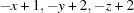; (ii) 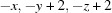.

**Fig. 1.**
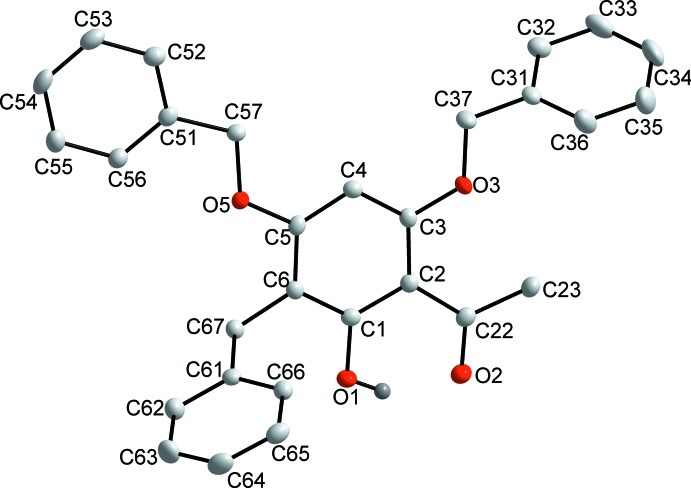
View of (I) (50% probability displacement ellipsoids). Selected hydrogen atoms have been omitted.

**Fig. 2.**
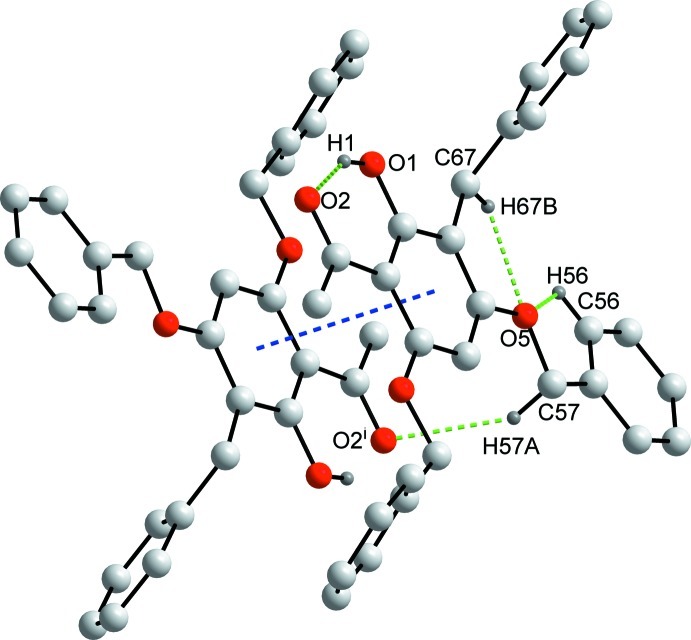
Inter- and Intramolecular hydrogen bond interactions along with π···π interactions are illustrated by dashed bonds. Symmetry code (i) -x + 1, -y + 2, -z + 2. Non-relavent hydrogen atoms have been omitted for clarity.

**Fig. 3.**
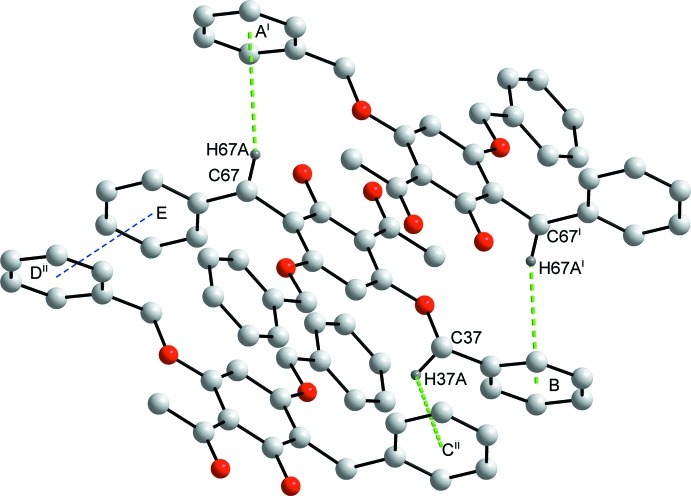
H···π interactions of I as illustrated by dashed bonds.. Symmetry codes (i) 1 - x, 2 - y, 2 - z and (ii) -x, 2 - y, 2 - z. Non-relavent hydrogen atoms have been omitted.

**Fig. 4.**
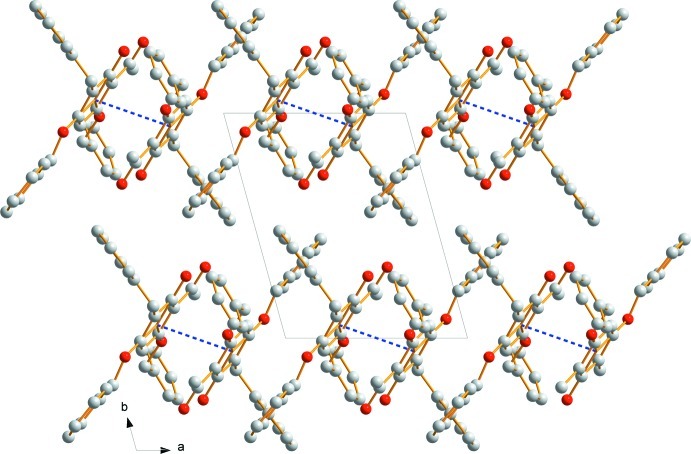
A packing diagram of I, illustrating a head-to-tail configuration as viewed along the c axis. The dashed bonds are shown to illustrate the π-stacking. Hydrogen atoms have been omitted.

## supplementary crystallographic information

### Comment

An interesting use of functionalized acetophenones is in the food industry as a flavourant (Burdock, 2010). Acetophenones are also one of the basic reactants for the synthesis of chalcones as discribed by Marais *et al.* (2005). As part of a study on the benzylation of nucleophilic polyphenols the structure of the undesired *C*-alkylation product (I) (Fig. 1) was obtained.

The backbone (C1—C6, C22, C23, C67, C37, O1—O3) including the benzyloxy group (C51 –C57, O5) of I was seen to be essentially planar with an *r.m.s.* deviation of 0.0311 Å, with a maximum deviation of 0.068 (1) Å for atom C23. The intramolecular hydrogen interactions of C56—H56···O5 and C67—H67B···O5 resulted in the benzyloxy group being planar with the backbone (Fig. 2). The angles of intersection between the planar backbone, the benzyl ring and the remaining aromatic ring of the benzyloxy group were found to be 70.45 (3)° and 59.55 (4)°. These angles are further stabilized by H···π interactions, C67—H67A···A^i^ (centriod C31—C36) and C37—H37A···C^ii^ (centriod C61—C66) with distances of 3.2894 (2) Å and 3.0139 (2) Å, respectively (Fig. 3). Additionally a very weak π···π interaction between rings D^ii^ (centriod C61—C66) and E (centriod C31—C36) with a distance of 4.5790 (2) Å was found.

A third intramolecular hydrogen interaction was observed for the oxygen (O2) of the ketone with the oxygen (O1) of the hydroxy group (Fig. 2). The last hydrogen bond is an intermolecular interaction of the methylene proton (H57A) with the ketone (O2) contributing to the head-to-tail packing of I, this is best seen in Fig. 2. As a result of this interaction, π-stacking is observed between the central aromatic rings with a distance of 3.6570 (2) Å, as further illustrated in Fig.4.

### Experimental

A mixture of 2,4,6-trihydroxy acetophenone (0.3 g, 1.78 mmol), benzyl chloride (0.431 ml, 3.75 mmol, 2.1 eq) and anhydrous potassium carbonate K_2_CO_3_ (0.493 g, 3.57 mmol, 2 eq) in dry DMF (15 ml) was heated at 80 °C for 1 hr with vigorous stirring. After the solid was filtered off, the filtrate was poured into 20 ml water, and extracted with Et_2_O. The extract was washed with water, brine, and dried over anhydrous MgSO_4_, and evaporated under reduced pressure. The crude product was purified by PTLC (*n*-Hexane:Acetone = 7:3, *R*_f_ 0.53) to afford 4,6-dibenzyloxy-2-hydroxy acetophenone as a yellow solid (0.153 g, 51%) and the title compound (I) as a yellow solid (0.133 g, 44%).

^1^H NMR (600 MHz, Acetone-d6) δ (p.p.m.): 7.58–7.11 (m, 15 H, 3xC_6_*H*_5_); 6.53 (s, 1 H, *H* {H_4_}); 5.27 (s, 4 H, OC*H*_2_Bn); 3.95 (s, 2 H, C*H*_2_Bn); 2.55 (s, 3 H, COC*H*_3_). ^13^C NMR (600 MHz, Acetone-d6) δ (p.p.m.): 203.4 (*C*OCH_3_); 163.7 (Ph {C5}); 162.7 (Ph {C3}); 161.4 (Ph {C1}); 141.6–125.4 (3x*C*_6_H_5_ {Bn}); 109.4 (Ph {C6}); 105.9 (Ph {C2}); 89.3 (Ph {C4}); 71.1 (CO*C*H_2_Bn); 70.0 (CO*C*H_2_Bn); 32.8 (CO*C*H_3_); 27.7 (C*C*H_2_Bn).

### Refinement

All H atoms were placed in geometrically idealized positions and constrained to ride on their parent atoms, with C—H = 0.95 Å and *U*_iso_(H) = 1.2 *U*_eq_(C) for aromatic H atoms, C—H = 0.98 Å and *U*_iso_(H) = 1.5 *U*_eq_(C) for methyl H atoms, C—H = 0.99 Å and *U*_iso_(H) = 1.2 *U*_eq_(C) for methylene H atoms and O—H = 0.84 Å and *U*_iso_(H) = 1.5 *U*_eq_(O) for hydroxyl H atoms. The H atoms of the methyl and hydroxyl groups were allowed to rotate with a fixed angle to fit the experimental electron density.

### Crystal data

**Table:**

C_29_H_26_O_4_	*Z* = 2
*M**_r_* = 438.5	*F*(000) = 464
Triclinic, *P*1	*D*_x_ = 1.294 Mg m^−^^3^
Hall symbol: -P 1	Mo *K*α radiation, λ = 0.71073 Å
*a* = 8.2349 (5) Å	Cell parameters from 5873 reflections
*b* = 10.5411 (7) Å	θ = 2.4–28.1°
*c* = 13.5115 (9) Å	µ = 0.09 mm^−^^1^
α = 93.412 (3)°	*T* = 100 K
β = 94.139 (3)°	Plate, colourless
γ = 105.102 (3)°	0.39 × 0.12 × 0.03 mm
*V* = 1125.66 (13) Å^3^	

### Data collection

**Table:**

Bruker X8 APEXII 4K KappaCCD diffractometer	5521 independent reflections
Radiation source: sealed tube	4071 reflections with *I* > 2σ(*I*)
Graphite monochromator	*R*_int_ = 0.032
Detector resolution: 512 pixels mm^-1^	θ_max_ = 28.3°, θ_min_ = 2.0°
φ and ω scans	*h* = −10→9
Absorption correction: multi-scan (*SADABS*; Bruker, 2004)	*k* = −14→13
*T*_min_ = 0.968, *T*_max_ = 0.997	*l* = −17→17
22325 measured reflections	

### Refinement

**Table:**

Refinement on *F*^2^	0 restraints
Least-squares matrix: full	H-atom parameters constrained
*R*[*F*^2^ > 2σ(*F*^2^)] = 0.043	*w* = 1/[σ^2^(*F*_o_^2^) + (0.0463*P*)^2^ + 0.2817*P*] where *P* = (*F*_o_^2^ + 2*F*_c_^2^)/3
*wR*(*F*^2^) = 0.107	(Δ/σ)_max_ = 0.001
*S* = 1.03	Δρ_max_ = 0.31 e Å^−^^3^
5521 reflections	Δρ_min_ = −0.22 e Å^−^^3^
300 parameters	

### Special details

**Table:**

Geometry. All e.s.d.'s (except the e.s.d. in the dihedral angle between two l.s. planes) are estimated using the full covariance matrix. The cell e.s.d.'s are taken into account individually in the estimation of e.s.d.'s in distances, angles and torsion angles; correlations between e.s.d.'s in cell parameters are only used when they are defined by crystal symmetry. An approximate (isotropic) treatment of cell e.s.d.'s is used for estimating e.s.d.'s involving l.s. planes.

### Fractional atomic coordinates and isotropic or equivalent isotropic displacement parameters (Å^2^)

**Table:**

	*x*	*y*	*z*	*U*_iso_*/*U*_eq_	
C1	0.43123 (15)	1.16936 (12)	1.05832 (9)	0.0158 (3)	
C2	0.43202 (14)	1.13497 (12)	0.95515 (9)	0.0154 (3)	
C3	0.30811 (15)	1.01942 (12)	0.91394 (9)	0.0151 (3)	
C4	0.19200 (14)	0.94574 (12)	0.97185 (9)	0.0158 (3)	
H4	0.1099	0.8689	0.9431	0.019*	
C5	0.19676 (14)	0.98530 (12)	1.07249 (9)	0.0152 (3)	
C6	0.31383 (15)	1.09821 (12)	1.11786 (9)	0.0153 (3)	
C22	0.55812 (15)	1.21880 (12)	0.89903 (10)	0.0170 (3)	
C23	0.56585 (17)	1.19593 (13)	0.78917 (10)	0.0215 (3)	
H23A	0.6572	1.2653	0.7672	0.032*	
H23B	0.4581	1.1976	0.7541	0.032*	
H23C	0.5873	1.1099	0.7744	0.032*	
C31	0.22496 (15)	0.85555 (13)	0.66335 (9)	0.0194 (3)	
C32	0.27035 (17)	0.74395 (14)	0.63042 (11)	0.0254 (3)	
H32	0.2723	0.6771	0.6743	0.031*	
C33	0.3130 (2)	0.72889 (16)	0.53403 (12)	0.0343 (4)	
H33	0.3438	0.6518	0.5121	0.041*	
C34	0.3109 (2)	0.82508 (17)	0.46990 (12)	0.0418 (4)	
H34	0.3415	0.8151	0.404	0.05*	
C35	0.2642 (3)	0.93618 (17)	0.50191 (12)	0.0454 (5)	
H35	0.2619	1.0026	0.4577	0.054*	
C36	0.2208 (2)	0.95114 (15)	0.59800 (11)	0.0329 (4)	
H36	0.1879	1.0276	0.6193	0.039*	
C37	0.18315 (16)	0.87355 (13)	0.76859 (10)	0.0210 (3)	
H37A	0.0707	0.8907	0.7697	0.025*	
H37B	0.182	0.7934	0.8035	0.025*	
C51	−0.11328 (15)	0.72877 (12)	1.18251 (10)	0.0167 (3)	
C52	−0.25410 (16)	0.62077 (13)	1.16099 (10)	0.0213 (3)	
H52	−0.2897	0.5881	1.0937	0.026*	
C53	−0.34264 (17)	0.56061 (13)	1.23669 (11)	0.0250 (3)	
H53	−0.4384	0.4872	1.2212	0.03*	
C54	−0.29123 (17)	0.60767 (13)	1.33487 (11)	0.0247 (3)	
H54	−0.3526	0.5674	1.3868	0.03*	
C55	−0.15038 (17)	0.71347 (13)	1.35738 (10)	0.0241 (3)	
H55	−0.114	0.7449	1.4248	0.029*	
C56	−0.06189 (16)	0.77387 (13)	1.28112 (10)	0.0210 (3)	
H56	0.0345	0.8466	1.2969	0.025*	
C57	−0.02121 (15)	0.79066 (12)	1.09773 (9)	0.0167 (3)	
H57A	0.0447	0.7329	1.07	0.02*	
H57B	−0.1032	0.8025	1.0441	0.02*	
C61	0.25514 (15)	1.26065 (12)	1.25126 (9)	0.0171 (3)	
C62	0.23856 (17)	1.29554 (13)	1.35037 (10)	0.0237 (3)	
H62	0.2714	1.2461	1.401	0.028*	
C63	0.17508 (19)	1.40109 (14)	1.37632 (11)	0.0299 (3)	
H63	0.1636	1.4227	1.4443	0.036*	
C64	0.12837 (18)	1.47526 (14)	1.30383 (11)	0.0281 (3)	
H64	0.0845	1.5475	1.3217	0.034*	
C65	0.14603 (17)	1.44345 (13)	1.20536 (11)	0.0240 (3)	
H65	0.115	1.4943	1.1552	0.029*	
C66	0.20922 (15)	1.33692 (12)	1.17949 (10)	0.0194 (3)	
H66	0.2211	1.316	1.1115	0.023*	
C67	0.32122 (16)	1.14155 (12)	1.22660 (9)	0.0179 (3)	
H67A	0.4402	1.1616	1.255	0.022*	
H67B	0.2559	1.0666	1.2604	0.022*	
O1	0.54647 (11)	1.27487 (9)	1.10477 (7)	0.0203 (2)	
H1	0.6119	1.3115	1.0638	0.03*	
O2	0.66614 (11)	1.31535 (9)	0.94318 (7)	0.0221 (2)	
O3	0.31270 (10)	0.98499 (8)	0.81615 (6)	0.0183 (2)	
O5	0.08963 (10)	0.91597 (8)	1.13482 (6)	0.0175 (2)	

### Atomic displacement parameters (Å^2^)

**Table:**

	*U*^11^	*U*^22^	*U*^33^	*U*^12^	*U*^13^	*U*^23^
C1	0.0144 (5)	0.0145 (6)	0.0184 (7)	0.0044 (5)	0.0002 (5)	0.0005 (5)
C2	0.0146 (5)	0.0159 (6)	0.0166 (6)	0.0053 (5)	0.0023 (5)	0.0019 (5)
C3	0.0151 (5)	0.0172 (6)	0.0144 (6)	0.0069 (5)	0.0015 (5)	0.0017 (5)
C4	0.0146 (5)	0.0152 (6)	0.0174 (7)	0.0042 (5)	0.0006 (5)	0.0002 (5)
C5	0.0136 (5)	0.0171 (6)	0.0166 (7)	0.0059 (5)	0.0029 (5)	0.0043 (5)
C6	0.0164 (6)	0.0161 (6)	0.0147 (6)	0.0065 (5)	0.0010 (5)	0.0017 (5)
C22	0.0159 (6)	0.0158 (6)	0.0207 (7)	0.0062 (5)	0.0026 (5)	0.0034 (5)
C23	0.0237 (6)	0.0194 (6)	0.0206 (7)	0.0026 (5)	0.0074 (5)	0.0034 (5)
C31	0.0176 (6)	0.0210 (6)	0.0161 (7)	0.0004 (5)	−0.0009 (5)	−0.0019 (5)
C32	0.0279 (7)	0.0228 (7)	0.0239 (8)	0.0055 (6)	−0.0016 (6)	−0.0016 (6)
C33	0.0367 (8)	0.0356 (9)	0.0286 (9)	0.0099 (7)	0.0021 (7)	−0.0130 (7)
C34	0.0515 (10)	0.0480 (10)	0.0176 (8)	−0.0010 (8)	0.0087 (7)	−0.0076 (7)
C35	0.0760 (13)	0.0357 (9)	0.0200 (8)	0.0061 (9)	0.0027 (8)	0.0075 (7)
C36	0.0527 (10)	0.0259 (8)	0.0208 (8)	0.0129 (7)	−0.0005 (7)	0.0005 (6)
C37	0.0188 (6)	0.0218 (7)	0.0182 (7)	−0.0010 (5)	0.0005 (5)	−0.0022 (5)
C51	0.0160 (6)	0.0157 (6)	0.0203 (7)	0.0067 (5)	0.0034 (5)	0.0039 (5)
C52	0.0211 (6)	0.0182 (6)	0.0238 (7)	0.0041 (5)	0.0016 (5)	0.0014 (5)
C53	0.0220 (6)	0.0177 (6)	0.0345 (8)	0.0022 (5)	0.0061 (6)	0.0054 (6)
C54	0.0285 (7)	0.0213 (7)	0.0284 (8)	0.0088 (6)	0.0137 (6)	0.0109 (6)
C55	0.0317 (7)	0.0227 (7)	0.0196 (7)	0.0082 (6)	0.0066 (6)	0.0039 (6)
C56	0.0227 (6)	0.0188 (6)	0.0205 (7)	0.0028 (5)	0.0039 (5)	0.0032 (5)
C57	0.0157 (6)	0.0156 (6)	0.0174 (7)	0.0017 (5)	0.0016 (5)	0.0001 (5)
C61	0.0153 (6)	0.0157 (6)	0.0180 (7)	0.0001 (5)	0.0022 (5)	0.0000 (5)
C62	0.0291 (7)	0.0227 (7)	0.0190 (7)	0.0058 (6)	0.0029 (5)	0.0021 (5)
C63	0.0416 (8)	0.0260 (7)	0.0230 (8)	0.0094 (6)	0.0105 (6)	−0.0022 (6)
C64	0.0322 (8)	0.0185 (7)	0.0355 (9)	0.0078 (6)	0.0120 (6)	0.0006 (6)
C65	0.0229 (7)	0.0189 (7)	0.0300 (8)	0.0041 (5)	0.0036 (6)	0.0055 (6)
C66	0.0188 (6)	0.0199 (6)	0.0181 (7)	0.0025 (5)	0.0030 (5)	0.0011 (5)
C67	0.0196 (6)	0.0181 (6)	0.0157 (7)	0.0044 (5)	0.0010 (5)	0.0013 (5)
O1	0.0191 (4)	0.0186 (5)	0.0192 (5)	−0.0016 (3)	0.0028 (4)	−0.0012 (4)
O2	0.0206 (5)	0.0203 (5)	0.0223 (5)	−0.0003 (4)	0.0031 (4)	0.0018 (4)
O3	0.0182 (4)	0.0204 (5)	0.0131 (5)	0.0000 (3)	0.0027 (3)	−0.0016 (4)
O5	0.0176 (4)	0.0169 (4)	0.0157 (5)	−0.0001 (3)	0.0033 (3)	0.0016 (3)

### Geometric parameters (Å, º)

**Table:**

C1—O1	1.3462 (14)	C37—H37B	0.99
C1—C6	1.3966 (17)	C51—C56	1.3852 (18)
C1—C2	1.4202 (17)	C51—C52	1.3962 (17)
C2—C3	1.4228 (16)	C51—C57	1.5064 (17)
C2—C22	1.4660 (17)	C52—C53	1.3870 (19)
C3—O3	1.3551 (15)	C52—H52	0.95
C3—C4	1.3867 (17)	C53—C54	1.384 (2)
C4—C5	1.3931 (17)	C53—H53	0.95
C4—H4	0.95	C54—C55	1.3843 (19)
C5—O5	1.3661 (14)	C54—H54	0.95
C5—C6	1.3970 (16)	C55—C56	1.3938 (18)
C6—C67	1.5046 (17)	C55—H55	0.95
C22—O2	1.2499 (15)	C56—H56	0.95
C22—C23	1.4983 (18)	C57—O5	1.4339 (14)
C23—H23A	0.98	C57—H57A	0.99
C23—H23B	0.98	C57—H57B	0.99
C23—H23C	0.98	C61—C66	1.3885 (18)
C31—C32	1.3829 (19)	C61—C62	1.3933 (18)
C31—C36	1.384 (2)	C61—C67	1.5217 (18)
C31—C37	1.4997 (18)	C62—C63	1.3846 (19)
C32—C33	1.384 (2)	C62—H62	0.95
C32—H32	0.95	C63—C64	1.383 (2)
C33—C34	1.375 (2)	C63—H63	0.95
C33—H33	0.95	C64—C65	1.380 (2)
C34—C35	1.380 (2)	C64—H64	0.95
C34—H34	0.95	C65—C66	1.3920 (19)
C35—C36	1.382 (2)	C65—H65	0.95
C35—H35	0.95	C66—H66	0.95
C36—H36	0.95	C67—H67A	0.99
C37—O3	1.4454 (14)	C67—H67B	0.99
C37—H37A	0.99	O1—H1	0.84
			
O1—C1—C6	115.78 (11)	C56—C51—C52	118.79 (12)
O1—C1—C2	120.94 (11)	C56—C51—C57	122.41 (11)
C6—C1—C2	123.28 (11)	C52—C51—C57	118.80 (11)
C1—C2—C3	116.48 (11)	C53—C52—C51	120.78 (13)
C1—C2—C22	118.76 (11)	C53—C52—H52	119.6
C3—C2—C22	124.76 (11)	C51—C52—H52	119.6
O3—C3—C4	122.45 (11)	C54—C53—C52	119.87 (12)
O3—C3—C2	116.19 (10)	C54—C53—H53	120.1
C4—C3—C2	121.34 (11)	C52—C53—H53	120.1
C3—C4—C5	119.44 (11)	C53—C54—C55	119.98 (12)
C3—C4—H4	120.3	C53—C54—H54	120
C5—C4—H4	120.3	C55—C54—H54	120
O5—C5—C4	122.82 (11)	C54—C55—C56	120.00 (13)
O5—C5—C6	114.76 (11)	C54—C55—H55	120
C4—C5—C6	122.40 (11)	C56—C55—H55	120
C1—C6—C5	117.01 (11)	C51—C56—C55	120.58 (12)
C1—C6—C67	119.98 (11)	C51—C56—H56	119.7
C5—C6—C67	122.97 (11)	C55—C56—H56	119.7
O2—C22—C2	119.75 (11)	O5—C57—C51	108.42 (10)
O2—C22—C23	116.79 (11)	O5—C57—H57A	110
C2—C22—C23	123.46 (11)	C51—C57—H57A	110
C22—C23—H23A	109.5	O5—C57—H57B	110
C22—C23—H23B	109.5	C51—C57—H57B	110
H23A—C23—H23B	109.5	H57A—C57—H57B	108.4
C22—C23—H23C	109.5	C66—C61—C62	117.80 (12)
H23A—C23—H23C	109.5	C66—C61—C67	123.21 (11)
H23B—C23—H23C	109.5	C62—C61—C67	118.99 (12)
C32—C31—C36	118.94 (13)	C63—C62—C61	121.13 (13)
C32—C31—C37	120.53 (12)	C63—C62—H62	119.4
C36—C31—C37	120.53 (12)	C61—C62—H62	119.4
C31—C32—C33	120.51 (14)	C64—C63—C62	120.33 (13)
C31—C32—H32	119.7	C64—C63—H63	119.8
C33—C32—H32	119.7	C62—C63—H63	119.8
C34—C33—C32	120.27 (15)	C65—C64—C63	119.43 (13)
C34—C33—H33	119.9	C65—C64—H64	120.3
C32—C33—H33	119.9	C63—C64—H64	120.3
C33—C34—C35	119.57 (15)	C64—C65—C66	120.09 (13)
C33—C34—H34	120.2	C64—C65—H65	120
C35—C34—H34	120.2	C66—C65—H65	120
C34—C35—C36	120.26 (16)	C61—C66—C65	121.21 (12)
C34—C35—H35	119.9	C61—C66—H66	119.4
C36—C35—H35	119.9	C65—C66—H66	119.4
C35—C36—C31	120.44 (15)	C6—C67—C61	116.24 (11)
C35—C36—H36	119.8	C6—C67—H67A	108.2
C31—C36—H36	119.8	C61—C67—H67A	108.2
O3—C37—C31	106.67 (10)	C6—C67—H67B	108.2
O3—C37—H37A	110.4	C61—C67—H67B	108.2
C31—C37—H37A	110.4	H67A—C67—H67B	107.4
O3—C37—H37B	110.4	C1—O1—H1	109.5
C31—C37—H37B	110.4	C3—O3—C37	118.66 (9)
H37A—C37—H37B	108.6	C5—O5—C57	118.65 (9)
			
O1—C1—C2—C3	178.13 (11)	C37—C31—C36—C35	178.08 (14)
C6—C1—C2—C3	−1.90 (18)	C32—C31—C37—O3	114.05 (13)
O1—C1—C2—C22	−1.70 (17)	C36—C31—C37—O3	−65.12 (16)
C6—C1—C2—C22	178.27 (11)	C56—C51—C52—C53	0.88 (19)
C1—C2—C3—O3	−178.13 (10)	C57—C51—C52—C53	179.95 (12)
C22—C2—C3—O3	1.69 (18)	C51—C52—C53—C54	−0.1 (2)
C1—C2—C3—C4	0.70 (17)	C52—C53—C54—C55	−0.9 (2)
C22—C2—C3—C4	−179.48 (12)	C53—C54—C55—C56	1.0 (2)
O3—C3—C4—C5	178.61 (11)	C52—C51—C56—C55	−0.74 (19)
C2—C3—C4—C5	−0.14 (18)	C57—C51—C56—C55	−179.78 (12)
C3—C4—C5—O5	−178.20 (11)	C54—C55—C56—C51	−0.2 (2)
C3—C4—C5—C6	0.70 (18)	C56—C51—C57—O5	−14.90 (16)
O1—C1—C6—C5	−177.61 (11)	C52—C51—C57—O5	166.07 (11)
C2—C1—C6—C5	2.42 (18)	C66—C61—C62—C63	1.32 (19)
O1—C1—C6—C67	0.22 (17)	C67—C61—C62—C63	−178.38 (13)
C2—C1—C6—C67	−179.75 (11)	C61—C62—C63—C64	−0.7 (2)
O5—C5—C6—C1	177.20 (10)	C62—C63—C64—C65	−0.2 (2)
C4—C5—C6—C1	−1.78 (18)	C63—C64—C65—C66	0.5 (2)
O5—C5—C6—C67	−0.56 (17)	C62—C61—C66—C65	−1.04 (18)
C4—C5—C6—C67	−179.54 (11)	C67—C61—C66—C65	178.65 (12)
C1—C2—C22—O2	2.78 (18)	C64—C65—C66—C61	0.2 (2)
C3—C2—C22—O2	−177.04 (12)	C1—C6—C67—C61	76.07 (15)
C1—C2—C22—C23	−177.02 (12)	C5—C6—C67—C61	−106.24 (14)
C3—C2—C22—C23	3.16 (19)	C66—C61—C67—C6	−7.73 (17)
C36—C31—C32—C33	0.8 (2)	C62—C61—C67—C6	171.96 (11)
C37—C31—C32—C33	−178.39 (13)	C4—C3—O3—C37	5.21 (17)
C31—C32—C33—C34	0.1 (2)	C2—C3—O3—C37	−175.98 (11)
C32—C33—C34—C35	−0.7 (3)	C31—C37—O3—C3	−177.49 (10)
C33—C34—C35—C36	0.4 (3)	C4—C5—O5—C57	6.31 (17)
C34—C35—C36—C31	0.5 (3)	C6—C5—O5—C57	−172.67 (10)
C32—C31—C36—C35	−1.1 (2)	C51—C57—O5—C5	174.63 (10)

### Hydrogen-bond geometry (Å, º)

Cg1 and Cg3 are the centroids of the C1–C6 and C31–C36 rings, respectively.

**Table:**

*D*—H···*A*	*D*—H	H···*A*	*D*···*A*	*D*—H···*A*
C56—H56···O5	0.95	2.39	2.7282 (15)	101
C67—H67*B*···O5	0.99	2.34	2.7882 (15)	106
O1—H1···O2	0.84	1.72	2.4744 (13)	148
C57—H57*A*···O2^i^	0.99	2.58	3.4592 (16)	148
C23—H23*B*···*Cg*3^ii^	0.98	2.83	3.6790 (16)	145
C57—H57*B*···*Cg*1^ii^	0.99	2.64	3.5106 (14)	146

Symmetry codes: (i) −*x*+1, −*y*+2, −*z*+2; (ii) −*x*, −*y*+2, −*z*+2.
